# Genomic footprints of bottleneck in landlocked salmon population

**DOI:** 10.1038/s41598-023-34076-0

**Published:** 2023-04-25

**Authors:** Sankar Subramanian, Manoharan Kumar

**Affiliations:** Centre for Bioinnovation, School of Science, Technology, and Engineering, The University of the Sunshine Coast, 1 Moreton Parade, Petrie, Moreton Bay, QLD 4502 Australia

**Keywords:** Evolutionary genetics, Population genetics, Computational biology and bioinformatics, Evolution, Genetics, Zoology, Ecology

## Abstract

At the end of the last ice age, several Atlantic salmon populations got caught up in the lakes and ponds of the Northern Hemisphere. Occasionally, the populations also got locked when the flow of rivers terminated from reaching the sea due to land upheaval. Therefore, the pattern of evolution shaping the landlocked salmon populations is different from the other anadromous salmons, which migrate between the sea and rivers. According to the theories of population genetics, the effect of genetic drift is expected to be more pronounced in the former compared to the latter. Here we examined this using the whole genome data of landlocked and anadromous salmon populations of Norway. Our results showed a 50–80% reduction in the genomic heterozygosity in the landlocked compared to anadromous salmon populations. The number and total size of the runs of homozygosity (RoH) segments of landlocked salmons were two to eightfold higher than those of their anadromous counterparts. We found the former had a higher ratio of nonsynonymous-to-synonymous diversities than the latter. The investigation also revealed a significant elevation of homozygous deleterious Single Nucleotide Variants (SNVs) in the landlocked salmon compared to the anadromous populations. All these results point to a significant reduction in the population size of the landlocked salmons. This process of reduction might have started recently as the phylogeny revealed a recent separation of the landlocked from the anadromous population. Previous studies on terrestrial vertebrates observed similar signatures of a bottleneck when the populations from Island and the mainland were compared. Since landlocked waterbody such as ponds and lakes are geographically analogous to Islands for fish populations, the findings of this study suggest the similarity in the patterns of evolution between the two.

## Introduction

The evolution of vertebrate populations on islands is quite different from that of those living on the mainland. Previous studies observed a reduction in the body sizes of island populations for most of the vertebrates, but an increase was reported in a few small animals^1,2^. This was called Foster’s rule or the Island effect. While the dwarfism was owing to the limited resource availability, the absence of predators was thought to be the reason for gigantism. On the other hand, studies based on molecular markers such as allozymes, microsatellites, and mitochondrial sequence data found a reduction in heterozygosity in island populations compared to their mainland relatives^3–10^. This could be due to the reduction in the effective population size that is forced by the limited availability of space in islands. The heterozygosity is determined by the effective population size and mutation rate^11^. Since the mutation rate does not change significantly between the Island and mainland populations of the same species, the decline in the population size leads to a proportional reduction in the heterozygosity. Apart from heterozygosity, recent studies based on whole genome data found a much higher number of runs of homozygosity (RoH) segments in Island populations than their mainland counterparts^6,7,12^. For example, the RoH segments constitute 23% of the mammoth genome from Wrangel Island, but these segments comprise only 0.83% of the mainland European mammoth^7^.

The accumulation of deleterious mutations is also a hallmark of population bottleneck, and therefore a number of studies compared the deleterious mutation load between the Island and mainland populations^6–8,12–15^. Most of these studies used the ratio of divergence or diversity at nonsynonymous and synonymous sites (dN/dS) to quantify the mutational load and found that this ratio was much higher in the island population than in their mainland counterparts. For instance, a previous study using 70 phylogenetically independent comparisons of populations from the island and mainland taxa showed that the dN/dS ratios of the former were significantly higher than those of the latter^15^. Using whole genome data from human populations found a much higher proportion of deleterious SNVs in Greenland populations compared to mainland Europeans^13^. Furthermore, whole genome-based studies comparing Island and mainland fox^8^ and kakapo^6^ populations observed similar results. These studies also observed a much higher proportion of homozygous deleterious SNVs in Island populations than the mainland ones.

During the last ice age, salmon populations from the Northern Oceans were translocated to the water bodies such as ponds and lakes in Europe and Northern America and were eventually got locked up after the glacial epoch^16–18^. In a different scenario, salmon populations had also become landlocked when a group of anadromous salmons prevented from reaching back to the sea when the river got terminated. A perfect example of this is the Trongfoss waterfall, which is part of the river Namsen of Norway that was isolated after the land upheaval^19^. The salmons in this river became landlocked and potentially descended from the anadromous population before the isolation. Hence this was reported to be the only river-living landlocked salmons as others survived in large lakes and ponds^19^. A previous study using over 6000 SNVs showed that the landlocked salmons in the river Namsen and the lake Byglandsfjorden had lower diversity than their North American and European anadromous counterparts^20^. Another study using pooled genome sequencing identified genomic regions that showed reduced diversity in the two groups of salmons^17^. Although the diversity estimates of landlocked salmons are known, many other important genomic signatures, such as the length of RoH, dN/dS ratio, and the accumulation of deleterious homozygous SNVs, could provide evidence for the potential bottleneck that could have occurred in landlocked salmons. Therefore, it is important to examine these genomic footprints in the landlocked populations and compare them with those of anadromous salmons. Since the aquatic animal populations in landlocked waterbodies are isolated from their counterparts in the ocean, this scenario is similar to the isolation of terrestrial animal populations in Islands from their mainland populations. Therefore, the same Island rule will hold true for the landlocked salmon, and the forces of evolution shaping these salmon populations are expected to be similar. Therefore, we examined this by comparing the whole genome heterozygosity, runs of homozygosity (RoH), and nonsynonymous and deleterious SNVs of a landlocked and five anadromous salmon populations of Norway. We also examined the phylogeny of the six populations to understand the relationship between the landlocked and anadromous salmon populations.

## Materials and methods

### Genome data

The whole genome raw sequence data in the fastq format for five landlocked salmons were available from a previous study^21^. For comparison, we obtained the whole genome data for 24 anadromous salmons belonging to five locations that were selected in order to include those representing a wide geographic area of Norway. The landlocked salmons were from an enclosed part of the river Namsen with a surface area of 12 km^2^. For comparison, we included five anadromous salmons from the Namsen river that was open to the sea. Additionally, five anadromous salmons from Southern Norway (Suldalslaagen river), five from Northern Norway (Tana river), five from the Baltic Sea, and four from the White Sea were included. Furthermore, we included the reference genome data (Sal_tru 1.1) of river trout (*Salmo trutta*) to use as the outgroup (see Supplementary Fig. S1 and Supplementary Table S1).

### Bioinformatic data processing

The *fastq* reads from 29 complete genomes were mapped to the Salmon reference genome (build Ssal v3.1) using the *bwa* aligner^22^. The mapped reads in the sequencing alignment mapped (SAM) format was converted to binary alignment/mapped (BAM) format using *Samtools*^23^. The aligned reads were then sorted based on the chromosomal positions, and then the PCR duplicates were removed using the *Picard* tool (https://broadinstitute.github.io/picard/). The genotypes for all chromosomal positions were called using *Samtools*. All 29 vcf files were merged into one file, and this was done for each chromosome. Finally, we filtered biallelic variant sites using an in-house awk script, which resulted in 41 million SNVs. In addition, we also estimated the average read depth for each sample using the “depth” module of the software *Samtool*. The total number of sites covered was calculated for each sample using an in-house script. The number of runs of homozygosity segments was estimated using the *plink* software^24^ with following parameter (–geno 0.01 –homozyg –homozyg-window-het 0 –maf 0.05). We used the whole genome data of the river trout to determine the direction of mutational change and to identify the derived alleles.

### Phylogenetic analysis

To examine the phylogenetic relationship using the genome data, we first estimated the genetic distances of all pairwise combinations of six salmon populations and the outgroup (Salmo trutta). We then used the program MEGA^25^ to compute the Neighbour-Joining tree. For generating bootstrap replicates, we randomly sampled SNVs from the genome data and created 500 pseudoreplicates. This was automated using an in-house Perl script, which also called on the program MEGA-CC^26^ to create an NJ tree for each pseudoreplicate dataset. All trees were then combined and fed to the program RaxML^27^ to compute bipartisan bootstrap scores for each node. Finally, the program FigTree (http://tree.bio.ed.ac.uk/software/figtree/) was used to draw the tree, and the bootstrap scores were displayed on each node. The genome sequence of *Salmo trutta* was used as the root for the salmon tree.

### Population genetic analysis

The heterozygosity (*H*) is the product of mutation rate (*μ*) and effective population size (*N*_*e*_) i.e., *H* = 4*N*_*e*_*μ*^28^. Recently, using the whole genome data on Atlantic herring^29^, Siamese fighter fish^30^, and Malawi cichlid fish^31^, three studies estimated the mutation rates in these fish to be 2.0 × 10^−9^, 3.5 × 10^−9^ and 3.8 × 10^−9^ per site per generation respectively. Although these estimates were obtained from widely different fish species, their rates were very similar (2.0–3.8 × 10^−9^). Hence, we used a middle value of 3.0 × 10^−9^ as the potential mutation rate of salmons and estimated the effective population size by rearranging the formula *N*_*e*_ = *H*/4*μ*.

It is well-known that synonymous mutations or SNVs change the nucleotide but do not change the amino acid coded by the codon due to codon degeneracy^32^. Therefore, synonymous mutations do not affect the protein structure and/or function as the amino acids are unaltered. Hence, synonymous SNVs are generally considered neutral as they are harmless and do not affect the fitness of the organism^32^. In contrast, nonsynonymous mutations or SNVs change the amino acid coded by the codons and hence, affect the structure and/or function of the proteins. Therefore, nonsynonymous mutations are under selection as they are harmful and affect the fitness of the organism. The ratio of these two reveals the proportion of harmful nonsynonymous SNVs present in a genome. A high proportion of this ratio suggests a high proportion of potentially deleterious nonsynonymous SNVs present in the genome. To identify synonymous and nonsynonymous SNVs, we annotated protein-coding regions using the tool *SNPeffect*^33^. The numbers of synonymous and nonsynonymous SNVs were divided by their respective number of synonymous and nonsynonymous sites to obtain the diversities at these sites. We estimated the ratio of the two was used to determine the accumulation of deleterious nonsynonymous SNVs in each genome.

In addition, we also used the GERP scores^34^, which were obtained from the data resource server Ensembl to detect deleterious SNVs. The GERP score for each chromosomal position was calculated using a multiple sequence alignment containing 90 fish genomes (https://ftp.ensembl.org/pub/release-108/bed/ensembl-compara/65_fish.gerp_constrained_element/gerp_constrained_elements.salmo_salar.bb). We used a threshold of > 4 to designate an SNV to be deleterious in nature. Using the genome annotations, we also identified the highly deleterious SNVs that cause premature termination or loss of function (LoF) of proteins. The keywords “stop_lost”, “stop_gained”, “start_lost”, “splice_donor” and “splice_acceptor” were used to detect the LoF SNVs.

The average number of LoF and deleterious SNVs per genome, along with the standard errors, were also estimated for each genome. The significance between the mean counts was determined using the Z test, and the statistical significance was determined using the software Z to P (http://vassarstats.net/tabs_z.html). A Pearson correlation coefficient was used to determine the strength of the correlation. The statistical significance of the correlation was determined by converting the correlation coefficient *r* to the normal deviation Z, and this was accomplished using the online software *r* to *P* (http://vassarstats.net/tabs_r.html).

## Results

### Phylogenetic relationship among salmon populations

**Figure 1:**
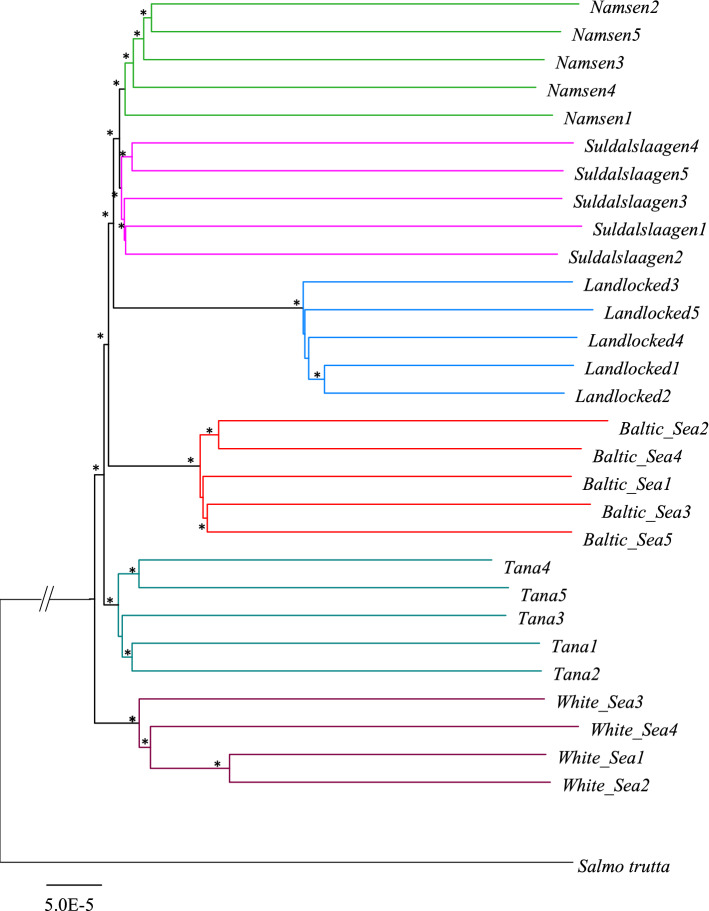
Phylogenetic relationship among the salmon populations from six locations in Norway. The brown trout was used as an outgroup to root the tree. The NJ tree was constructed using the whole genome data and the bootstrap confidence values were based on 500 replicates. The asterisk (*) denotes 100% bootstrap support. However, the nodes without an asterisk have > 80% but < 100% bootstrap support.

The genome data from 29 salmons belonging to six populations of Norway were used to infer the phylogenetic relationship between them. Figure 1 shows that populations in all six regions are monophyletic with 100% bootstrap support. The landlocked Namsen (Blanken) river salmon population forms a sister group with equidistance to the anadromous Namsen (Bjoera) and Suldalslaagen river populations. The population from the Baltic Sea (Torino) was closer to the southern populations than that from the northern Tana (Utsjoki) river. The White Sea (Keret) population was distantly related to the rest of the population. The genome of the brown trout was used to root the tree. In the following figures, the populations were ordered based on their phylogenetic relationship with the landlocked salmons.

### Genomic heterozygosity

**Figure 2:**
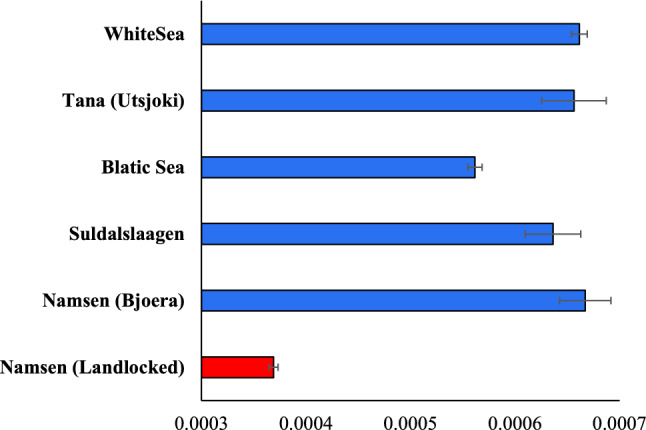
Genomic heterozygosities estimated for five anadromous and one landlocked population of Norway are shown. Error bars denote the standard error of the mean. The genomic diversity of the landlocked salmon population was significantly smaller than those of anadromous populations (at least, *P* < 0.0001).

We estimated the heterozygosity per site (see Supplementary Fig. S1 and Supplementary Table S1) using 41 million SNVs from the whole genomes of the six populations. This revealed that the nucleotide diversity of the landlocked salmon population was 0.00037 (Fig. 2). The diversity estimates for the anadromous salmons varied between 0.00056 and 0.00067. The genomic heterozygosity estimated for the landlocked salmon (Namsen_landlocked) population was significantly (at least, *P* < 0.0001, *Z* test) smaller than those observed for the five anadromous populations, including the one (Namsen_Bjoera) that was closely located to the former. The estimate for the anadromous Baltic Sea population was 50% higher than that of the landlocked population, and those obtained for other anadromous populations were 72–80% higher than that of the landlocked ones. Furthermore, except for the estimate of the Baltic Sea populations, the heterozygosity obtained for other anadromous populations was similar-statistically not different (*P* = 0.31).

### Runs of homozygosity (RoH)

**Figure 3:**
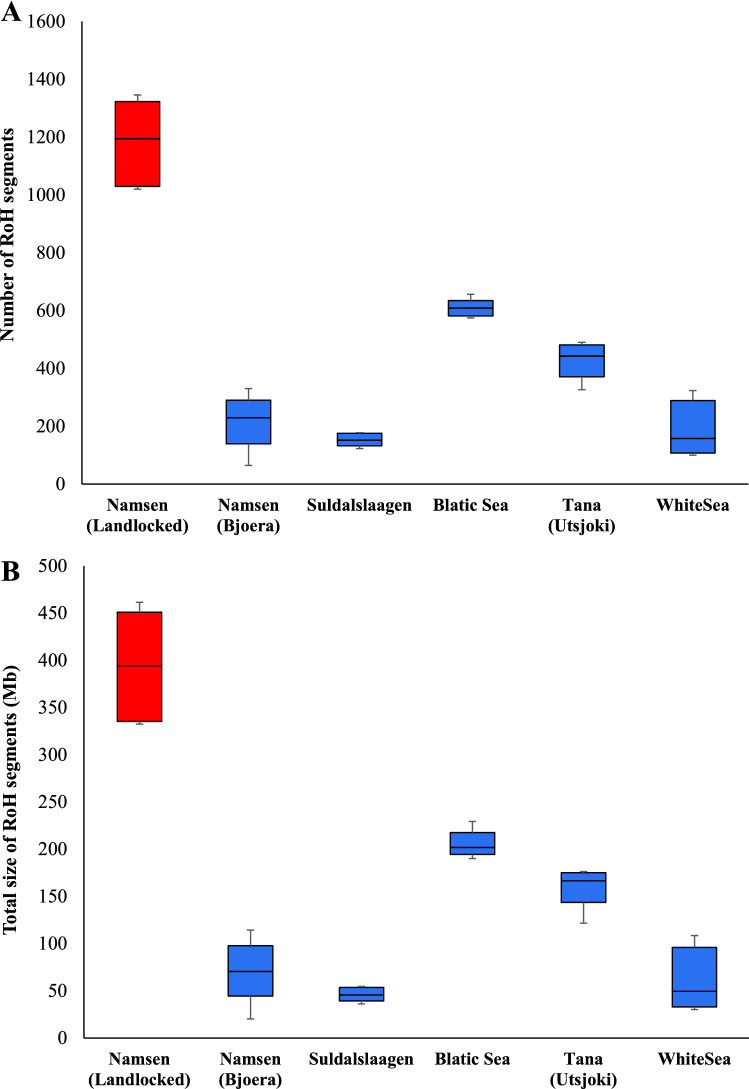
Box plot showing (**A**) the number and (**B**) the total size of runs of homozygous (RoH) segments in landlocked and anadromous salmon populations. The centre line denotes the median, the boundaries of the box represent the first and third quartiles, and the whiskers show the maximum and minimum values. The mean number of RoH of the landlocked salmon population was significantly higher than those of anadromous populations (at least, *P* = 0.0003). Similarly, the average size of RoH of the landlocked salmon population was significantly higher than those of anadromous populations (at least, *P* = 0.002).

To understand the level of homozygosity between landlocked and anadromous salmon populations, we investigated the number and size of RoH in each genome. We used a threshold of > 0.5 Mb to designate an RoH segment. Figure 3A shows that the mean number of RoH segments in landlocked salmons is 1180. The estimates for anadromous salmon populations range between 153 and 608. Therefore, the number of RoH segments in the landlocked population is approximately 1.9–7.7 times higher than those of the anadromous salmon populations, and the differences between them were highly significant (at least, *P* = 0.0003). We then compared the size of the RoH segments, which also revealed that the mean size of RoH segments in landlocked salmons was significantly (at least, *P* = 0.002) higher than those observed for anadromous salmons (Fig. 3B). The estimate for the former was 393 Mb and for the latter ranges between 46 and 205 Mb. Therefore, the mean size of RoH in landlocked salmons was approximately 1.9–8.5 times larger than those estimated for anadromous salmons.

### The ratio of the diversities at nonsynonymous and synonymous sites

**Figure 4:**
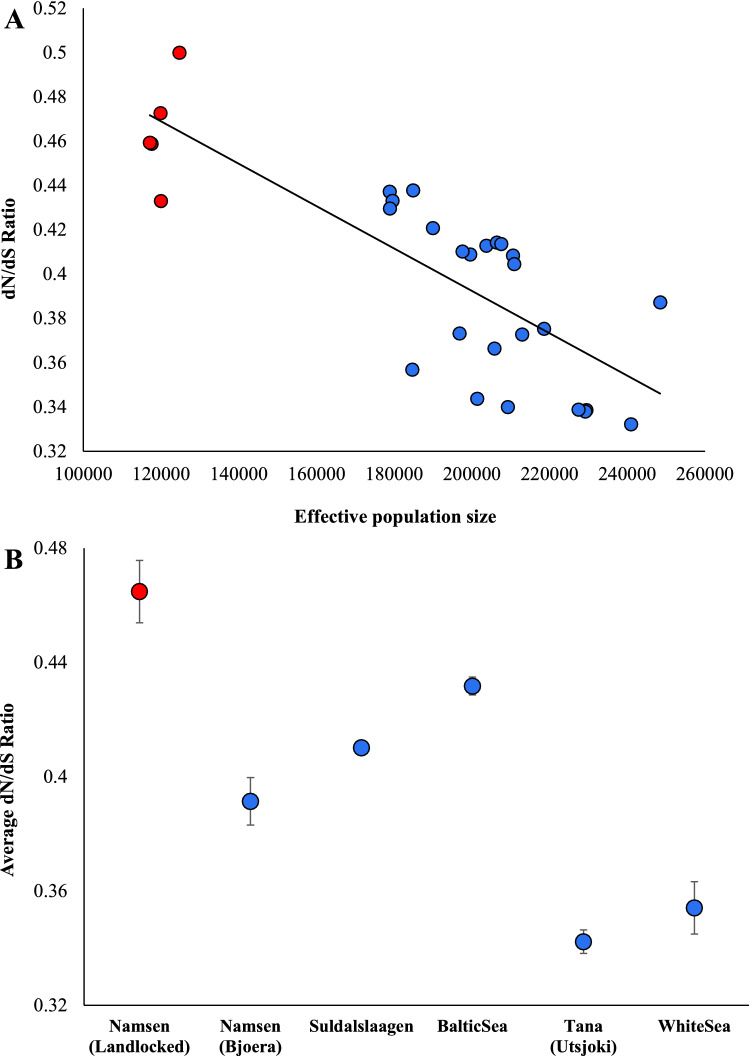
(**A**) The correlation between effective population size and the ratio of nonsynonymous and synonymous diversities (dN/dS). The relationship was highly significant (*r* = 0.79, *P* < 0.000001). The best-fitting regression line is shown. (**B**) The average dN/dS ratio was estimated for landlocked and anadromous salmon populations. Error bars show the standard error of the mean. The ratio observed for landlocked salmons was significantly higher than those of anadromous ones (at least, *P* = 0.0018).

To measure the accumulation of deleterious variants, we first used the ratio of nonsynonymous and synonymous variations (dN/dS) (see “Methods”). As explained in the methods, the dN/dS values show the proportion of potentially harmful nonsynonymous SNVs (nSNVs) in a genome, and a higher proportion suggests a higher accumulation of nSNVs. We then calculated the effective population size using the heterozygosity obtained from whole genome analysis (see “Methods”). We then plotted the dN/dS ratio against the effective population size estimated (Fig. 4A). The regression analysis revealed a highly significant (*r* = 0.79, *P* < 0.000001) negative correlation between the two variables. This suggests that individuals with small population sizes have high dN/dS ratios. Importantly, the dN/dS ratio of landlocked salmons was much higher than that of anadromous salmons, as the mean population size of the former (120,000) was much smaller than the latter (206,000). This has been clarified in Fig. 4B, which shows that the mean dN/dS ratio estimated for landlocked salmons was significantly (*P* = 0.0018) higher than those observed for the anadromous salmon populations.

**Figure 5:**
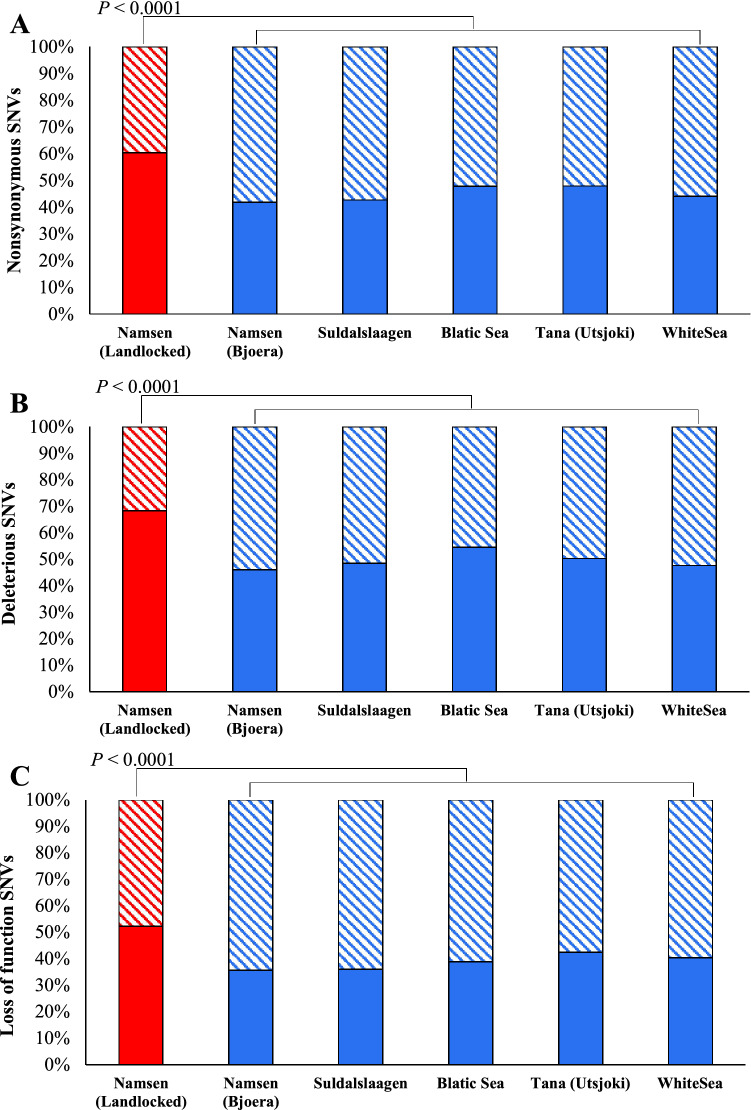
The stacked bar shows the proportions of homozygous (solid) and heterozygous (stripped) SNVs estimated for landlocked and anadromous salmon populations. (**A**) Nonsynonymous SNVs (**B**) Deleterious SNVs (**C**) Loss of function (LoF) SNVs. The number of homozygous SNVs in landlocked salmons was significantly higher than those estimated for the anadromous salmon, and this is true for the comparisons involving nonsynonymous, deleterious, and LoF SNVs (at least *P* < 0.0001).

We then calculated the number of homozygous and heterozygous nonsynonymous SNVs and plotted their proportions in stacked column graphs. Figure 5A reveals that the proportion of homozygous SNVs estimated for landlocked salmons was 60%, and for the anadromous salmons, this ranged between 42 and 48%. Furthermore, the average number of homozygous SNVs of the landlocked population (19,628) was significantly higher than those of anadromous populations (15,290–17,461) (at least, *P* < 0.0001). A similar analysis was performed using genome-wide deleterious SNVs, and we used the GERP score to identify deleterious SNVs (see “Methods”). This showed a much higher proportion (68%) of homozygous deleterious SNVs in landlocked salmons compared to those estimated for anadromous populations (46–55%) (Fig. 5B). The mean number of homozygous deleterious SNVs of the former (644) was significantly higher (at least, *P* < 0.0001) than those of the latter (457–536). Finally, the analysis using the loss of function (LoF) SNVs also revealed the same pattern (Fig. 5C). The landlocked populations had a much higher proportion of homozygous LoF SNVs (52%) than the proportions estimated for the anadromous populations (36–42%). As expected, the mean homozygous LoF SNV counts of the landlocked (1273) was significantly higher (at least, *P* < 0.0001) than those observed for the anadromous ones (954–1120).

## Discussion

Using the whole genome data from anadromous and landlocked salmon populations, we performed phylogenetic and population genomic analyses. The phylogeny of salmon populations suggests that the landlocked salmon separated from the common ancestor of the anadromous populations that currently live in the Namsen (Bjoera) and Suldalslaagen rivers. Hence, the reduction in the population size might have occurred more recently after the separation of the landlocked populations. This is in contrast with other landlocked salmons found in lakes and ponds, which have potentially been locked up after the ice ages^19^. The result from the phylogenetic analysis also informs that the genetic relationship is in concordance with the geographical proximity of the salmon populations.

The whole genome-based population genetic analysis conducted in this study provided four lines of evidence of severe bottleneck in the landlocked salmons. First, we observed a much-reduced genomic diversity in landlocked populations compared to anadromous ones. The result from our whole genome data analysis confirms previous studies based on a few microsatellite and allozyme data from a few loci^35,36^. Furthermore, this result is similar to earlier studies on many terrestrial mammals and birds^3,4,6–10,12,13^. The heterozygosity estimated for the Island populations were 20% to 84-fold (e.g., Island fox) higher than those observed for their respective mainland counterparts^8^. Second, the number and total size of RoH estimated for the landlocked genomes were two eightfold higher than those of anadromous populations. Earlier studies comparing Wrangel Island and European mainland mammoth populations showed a several-fold increase in the RoH content of the former^7^. A similar observation was reported comparing the Steward Island and mainland New Zealand populations of kakapo^6^ and between the wolves of Isle Royale and mainland Minnesota^12^. These studies showed that the Island populations had higher proportions of both medium-sized (0.2–1 Mb) and long (> 2 Mb) RoH. While there was a significant number of medium-sized RoH in landlocked salmons (Fig. 3), only a very few (< 10) long RoH were observed. The latter suggests that there was no significant inbreeding in the landlocked populations. Because it has been shown that inbreeding produces long RoH, and on the contrary, the reduction in population size alone creates predominantly medium-sized RoH^37^.

Third, we showed a much higher dN/dS ratio for landlocked salmons than anadromous ones (Fig. 4). The dN/dS ratio suggests a higher proportion of deleterious nonsynonymous SNVs present in the former in comparison with the latter. This is because the effective population size of landlocked salmons was 58% smaller than their anadromous counterparts (120,000 vs. 206,000). Since genetic drift is high in small populations, natural selection is inefficient in removing deleterious nonsynonymous SNVs^38^. Therefore, the high accumulation of potentially harmful nonsynonymous SNVs further confirms the population bottleneck that occurred in the landlocked salmons. A number of previous studies have shown a much higher dN/dS ratio in the Island populations of terrestrial vertebrates compared to their mainland relatives^8,14,15,39^. Fourth, our findings revealed a much higher proportion of homozygous deleterious SNVs in landlocked salmons than in anadromous populations (Fig. 5). This suggests that a greater number of heterozygous deleterious SNVs are converted to the homozygous state due to strong genetic drift. In contrast, purifying selection prevents low-frequency SNVs from reaching high frequencies^38^, and hence heterozygous SNVs are not allowed to be converted to homozygous ones. Similar patterns were reported in Island foxes^8^, Isle Royale wolves^12^, and Greenland Inuit populations^13^ in comparison with their respective mainland cousins.

All results of this study point out that there was a significant reduction in the population size of the landlocked salmons after they had been captured in the land-encircled section of the Namsen river with a surface area of 12 km^2^. This is similar to those observed for the water-locked Island populations of terrestrial vertebrates. Therefore, we can predict that the pattern of evolutionary forces shaping the landlocked populations will be very similar to those operating on the terrestrial vertebrate populations living on the Islands.

## Supplementary Information

Supplementary Information.

## Data Availability

Raw read sequence data (ID: PRJEB38061) used in this study was obtained from the SRA database (https://www.ncbi.nlm.nih.gov/sra). The details of the accession numbers and metadata are given in the supplementary material.
