# Supplementary material for: Genomic footprints of bottleneck in landlocked salmon population
Source: Sci Rep. 2023 Apr 25;13:6706. doi: 10.1038/s41598-023-34076-0 (PMC10130149; doi:10.1038/s41598-023-34076-0)
Supplement: Supplementary file 1 — Supplementary Information. [file 41598_2023_34076_MOESM1_ESM.pdf]

Genomic footprints of bottleneck in landlocked salmon population

Sankar Subramanian* and Manoharan Kumar

[1]Centre for Bioinnovation, School of Science, Technology and Engineering, The University of the Sunshine Coast, Moreton Bay, QLD 4502, Australia

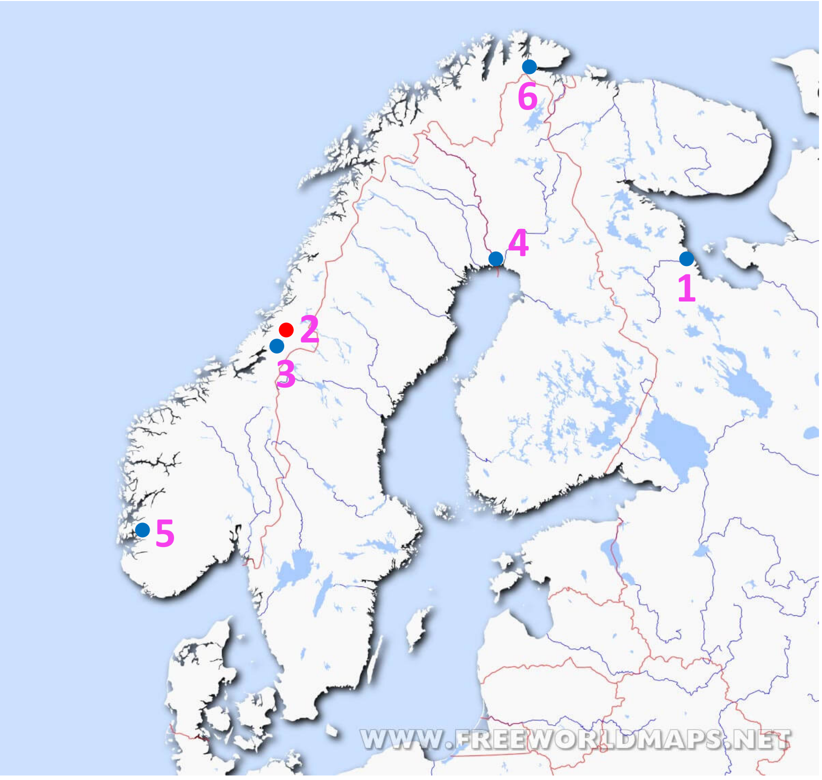

Figure S1. Locations of Landlocked (red circle) and anadromous (blue circles) salmon samples in Norway.

Table S1. Sample locations and their ID in the Sequence Read Archive database

SRA ID	Location name	Origin	Number
ERR4265446	Keret	White sea	1
ERR4265445	Keret	White sea	1
ERR8684049	Keret	White sea	1
ERR4265444	Keret	White sea	1
ERR8684051	Namsen_(Blanken)	Landlocked	2
ERR8684052	Namsen_(Blanken)	Landlocked	2
ERR4280257	Namsen_(Blanken)	Landlocked	2
ERR4280275	Namsen_(Blanken)	Landlocked	2
ERR8684055	Namsen_(Blanken)	Landlocked	2
ERR4280280	Namsen_(Bjoera)	Norway	3
ERR4280277	Namsen_(Bjoera)	Norway	3
ERR4280281	Namsen_(Bjoera)	Norway	3
ERR4280279	Namsen_(Bjoera)	Norway	3
ERR4280282	Namsen_(Bjoera)	Norway	3
ERR4280396	Tornio	Baltic	4
ERR4280394	Tornio	Baltic	4
ERR4280395	Tornio	Baltic	4
ERR4280397	Tornio	Baltic	4
ERR4280398	Tornio	Baltic	4
ERR4280475	Suldalslaagen	Norway	5
ERR4280474	Suldalslaagen	Norway	5
ERR4280477	Suldalslaagen	Norway	5
ERR4280478	Suldalslaagen	Norway	5
ERR4280480	Suldalslaagen	Norway	5
ERR8684031	Tana_(Utsjoki)	Norway	6
ERR8684032	Tana_(Utsjoki)	Norway	6
ERR8684033	Tana_(Utsjoki)	Norway	6
ERR8684034	Tana_(Utsjoki)	Norway	6
ERR8684037	Tana_(Utsjoki)	Norway	6
SAMEA7567002	Salmo trutta		
